# An Improved Pulse-Coupled Neural Network Model for Pansharpening

**DOI:** 10.3390/s20102764

**Published:** 2020-05-12

**Authors:** Xiaojun Li, Haowen Yan, Weiying Xie, Lu Kang, Yi Tian

**Affiliations:** 1 Faculty of Geomatics, Lanzhou Jiaotong University, Lanzhou 730070, China; xjli@mail.lzjtu.cn; 2 National-Local Joint Engineering Research Center of Technologies and Applications for National Geographic State Monitoring, Lanzhou 730070, China; 3 State Key Laboratory of Integrated Service Network, Xidian University, Xi’an 710071, China; wyxie@xidian.edu.cn; 4 State Key Laboratory of Resources and Environmental Information System, Institute of Geographical Sciences and Natural Resources Research, CAS, Beijing 100101, China; kangl@lreis.ac.cn; 5 Guangdong Provincial Key Laboratory of Robotics and Intelligent System, Shenzhen Institutes of Advanced Technology, CAS, Shenzhen 518172, China; yi.tian1@siat.ac.cn

**Keywords:** multispectral image, pansharpening, pulse-coupled neural network, high-resolution image, image fusion

## Abstract

Pulse-coupled neural network (PCNN) and its modified models are suitable for dealing with multi-focus and medical image fusion tasks. Unfortunately, PCNNs are difficult to directly apply to multispectral image fusion, especially when the spectral fidelity is considered. A key problem is that most fusion methods using PCNNs usually focus on the selection mechanism either in the space domain or in the transform domain, rather than a details injection mechanism, which is of utmost importance in multispectral image fusion. Thus, a novel pansharpening PCNN model for multispectral image fusion is proposed. The new model is designed to acquire the spectral fidelity in terms of human visual perception for the fusion tasks. The experimental results, examined by different kinds of datasets, show the suitability of the proposed model for pansharpening.

## 1. Introduction

Multispectral (MS) image is of great importance in typical remote sensing applications, such as land utilization [1], urban interpretation [2], urban monitoring [3], and scene understanding [4] tasks. However, owing to the physical constraints of the optical satellite sensor and its communication bandwidth, the spectral diversity and the high spatial resolution cannot be obtained at the same time. In other words, the finer spectral resolution is obtained at the cost of the coarser spatial resolution. Thus, pansharpening—which combines the narrow-band multispectral image with a broadband high spatial resolution panchromatic (PAN) image to acquire a high-resolution multispectral image—is desirable. The contributions of the pansharpening technology for remote sensing tasks mainly include change detection [5], feature extraction [6], land classification [7] and scene interpretation [8]. Detailed reviews of the pansharpening methods can be found in [9,10,11,12].

Most popular methods for pansharpening fall into two main groups, which are the component substitution (CS) methods and the multiresolution analysis (MRA) methods [9]. The CS methods mean that one of a component extracted from the multispectral image is substituted by using the PAN image. It consists of the methods, such as the intensity-hue-saturation method (IHS) [13,14], the principal component analysis method (PCA) [15,16], and the Gram–Schmidt (GS) method [17]. The MRA methods provide effective multiresolution decomposition tools to obtain spatial details from the high-resolution image, which could be injected into the resampled multispectral images. These algorithms include the decimated wavelet transform (DWT) [18], the undecimated wavelet transform (UDWT) [19], the “à trous” wavelet transform (ATWT) [20], the Laplacian pyramid (LP) [21], and the morphological pyramid [22]. More recently, several deep learning approaches have been proposed to address the fusion problem, which achieves the better fusion results through studying the training dataset. It consists of methods such as the convolutional neural networks method [23] and the pansharpening convolutional neural networks method [24]. Other pansharpening methods have been also proposed for the preprocessed steps or the misaligned data, such as the enhanced back-projection model [25] and the improved fusion model [26].

As a basic remote sensing problem, pansharpening is getting more attention along with the rapid development of the high-resolution satellite image. The platforms of Google Earth and Microsoft Bing are the most representative applications of pansharpening [27]. The main purpose of pansharpening is to inject the details from the higher resolution image into the lower ones so as to obtain the high-resolution multispectral image. Classical methods produce the injection coefficients either through global estimation or locally used rectangular windows. Obviously, global injection algorithms will inevitably result in more spectral distortion, because all the extracted details are treated without distinction. Local estimation of the injection coefficients performs better than global ones by considering the correlations of the neighborhood pixels. However, the local estimation areas for the injection procedure are always restricted in a fixed square window [28], which will provide frustrating results as the foreground and the background pixels appear in the same square. The mixed situation in a given square window is especially common in high-resolution remote sensing images, since the tiny objects are more likely available for the high-resolution images. Thus, the injection coefficients should be estimated in an irregular region rather than a square one. The pulse-coupled neural network (PCNN), which derives from the synchronization oscillation phenomenon of visual cortex neuron, has the ability to generate the irregular clustering regions. The synchronization oscillation phenomenon means that the neurons (corresponding to the pixels in an image) of a similar status will release the pulses at the same time.

The PCNN has shown great ability in image processing and pattern recognition applications [29]. More specifically, one of the most diffused uses of PCNN and its improved versions is in dealing with the image fusion problem, which includes m-PCNN [30], memristive PCNN [31], memristor-based PCNN [32], and shuffled frog leaping PCNN [33]. Though these models have commendable ability in the multi-focus image fusion and medical image fusion, they are difficult to apply directly in pansharpening. All these PCNN fusion models suggest that the input channels are similar and parallel, which lacks the considerations of details injection and spectral preservation. Obviously, the degree of spectral distortion is of the utmost importance in the pansharpening assessment of the remote sensing images. Therefore, the issue is unable to be directly addressed by traditional PCNNs. Cheng proposed a new adaptive dual-channel PCNN for fusing the infrared and visual images [34], which exhibits good fusion performance. Ma et al. also tried to address the fusion issues for infrared and visible images by using neural networks, such as the DDcGAN method [35], the FusionGAN method [36], and the detail-preserving adversarial learning method [37]. These models have attained very satisfactory results. Nonetheless, the application does not take the spectral channels into account. Shi et al. presented a novel PCNN based algorithm for remote sensing image fusion [38], which can achieve good fusion results for the multi-source remote sensing images. However, the fusion algorithm mainly aimed for image enhancement, and spectral fidelity is not under consideration.

To settle the issue, a pansharpening PCNN (PPCNN) model for multispectral image fusion is proposed in the paper. It can adaptively inject spatial details to the multispectral images in each iteration. Benefiting from the characteristic of the synchronous pulse emitting phenomenon of PPCNN, the detail injection estimation can be manipulated among the pixels which not only have similar values, but also similar neighborhoods. In addition, the proposed model is proved to coincide with the human visual perception, which results in the better visual inspection of fusion results. In the paper, the performance of the PPCNN has been well tested with five different high-resolution datasets. The results have been well assessed by both the spectral and spatial quality evaluation criterion. The results of the experiments indicate that the proposed multispectral fusion method is effective for pansharpening.

The rest of the article is organized as follows: Section 2 reviews the standard PCNN model in brief, and then proposes a novel PPCNN model. Section 3 is devoted to presenting a pansharpening method based on the proposed PPCNN model. Section 4 gives the experimental method. Experimental results and discussions are exhibited in Section 5, and Section 6 summarizes the work.

## 2. PCNN and PPCNN Models

To propose the new PPCNN model, the standard PCNN model is briefly introduced. Next, the proposed PPCNN is introduced. The improvements on the standard PCNN model are based on the practical demands of the pansharpening applications. The analysis and the implementation of the new model are also given in the section.

### 2.1. Standard PCNN Model

**Figure 1:**
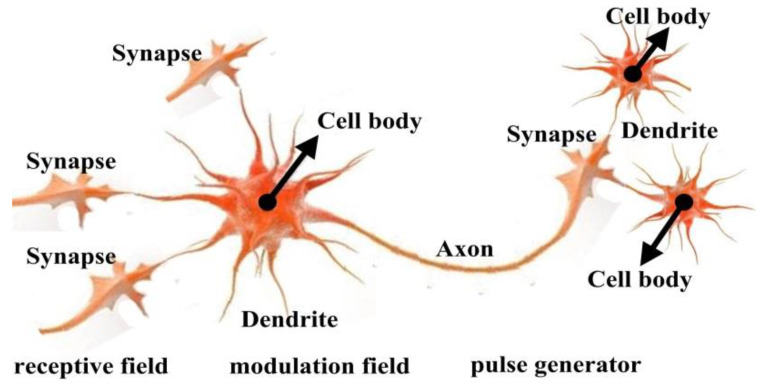
Structure of visual cortex neuron.

The brain, as is well-known, perceives the outside world through a complicated neural network of visual cortex neurons. The information of the real world is transmitted by thousands of neurons in the network. Each neuron consists of the cell body, the synapse, the axon and the dendrites. As shown in Figure 1, the cell body receives the electrical impulses from the synapses of other neurons via its dendrites. The membrane potential of the cell body rises as the neuron continues to be stimulated by other neurons. The electrical impulse is generated when the membrane potential is larger than its threshold. The threshold of current neuron changes in a nonlinear way after stimulation. On the other hand, the neuron also transmits electrical impulses to other neurons via the axon part.

**Figure 2:**
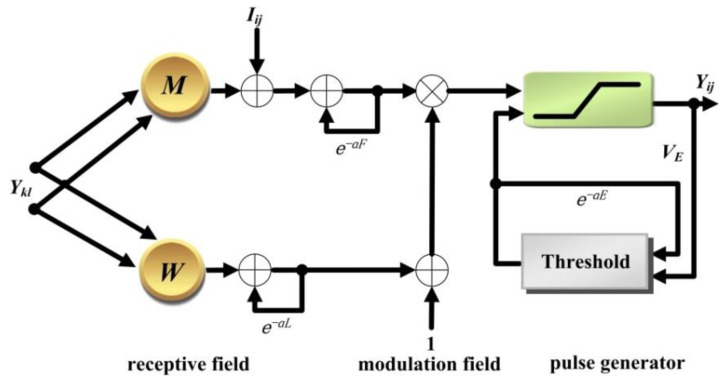
Structure of standard PCNN model.

As inspired by the structure of visual cortex neuron, Johnson et al. proposed the PCNN model [39]. PCNN, which imitates the mechanism of mammalian visual cortex, is a laterally connected neural network of two-dimensional neurons with no training. As shown in Figure 2, each neuron of the standard PCNN model is divided into three sequential parts: the receptive field, the modulation field, and the pulse generator. It can be mathematically described as follows [39,40]:

(1) $$F_{ij}[n]=e^{-\alpha_{F}}F_{ij}[n-1]+V_{F}\sum_{kl}M_{ijkl}Y_{kl}[n-1]+I_{ij}$$

(2) $$L_{ij}[n]=e^{-\alpha_{L}}L_{ij}[n-1]+V_{L}\sum_{kl}W_{ijkl}Y_{kl}[n-1]$$

(3) $$U_{ij}[n]=F_{ij}[n]\left(1+\beta L_{ij}[n]\right)$$

(4) $$Y_{ij}[n]=\{\begin{array}{ll} 1 & U_{ij}[n]>E_{ij}[n]\\ 0 & \mathrm{otherwise} \end{array}$$

(5) $$E_{ij}[n+1]=e^{-\alpha_{E}}E_{ij}[n]+V_{E}Y_{ij}[n]$$

In the PCNN model, the position of the neuron is denoted as a two-dimension symbol *ij* (*ij* means that the position of the neuron is at row *i*, column *j*), so as to be conveniently applied for an image. The neuron *kl* is defined as the neighboring neuron of the current neuron *ij*. The neuron *ij* receives the electrical impulses from the synapses of neighboring neurons *kl*, which simulates the functions of the dendrite part to receive the local stimulus via linking synapse *M* (or *W*). After the electrical impulses are gathered by the cell, the inputs are distinguished into two channels. One channel is the feeding input *F* and the other is the linking input *L*. The difference between them is that the feeding input *F* is influenced by the external stimulus *I*. In the modulation part, the internal activity *U* is designed to imitate the membrane potential of the cell body. The internal activity *U* is obtained by the coupling of *F* and weighted *L*, where the weighting factor is typically denoted as *β*. The pulse generator will produce the output pulse *Y*, if the dynamic threshold *E* is less than the internal activity *U*. Furthermore, *V_F_*, *V_L_* and *V_E_* indicate normalizing parameters. The parameters *α_F_*, *α_L_* and *α_E_* denote the exponential decay coefficients, which imitate the exponential attenuation characteristics over time.

### 2.2. Proposed PPCNN Model

**Figure 3:**
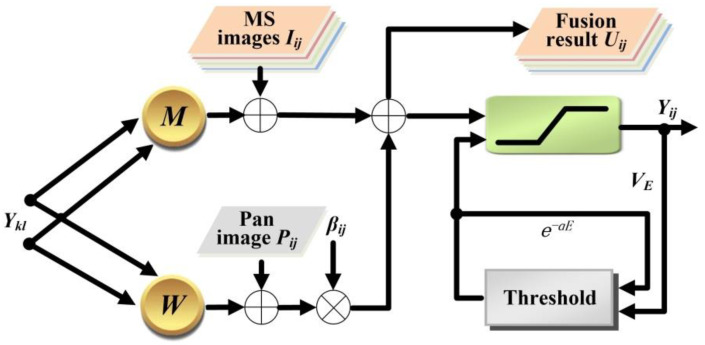
Structure of proposed PPCNN.

In order to make the PCNN model appropriate for pansharpening, an improved PCNN model is proposed, which is called PPCNN hereafter. For the convenience of the fusion task, the new model should be designed to have two external inputs, and its characteristic of synchronous pulse emitting should remain unchanged. The neuron structure of the improved model is shown in Figure 3. Compared with the standard PCNN, the PPCNN model has at least four advantages: (1) two external stimuli *I* and *P* are considered simultaneously for the convenience of fusion operations, (2) each neuron *ij* of PPCNN has its own weighting factor *β_ij_* rather than a uniform one in the standard PCNN model, (3) the internal activity *U*, which is composed of the sum of the feeding part *F* and the weighted linking part *L*, is considered as the final fusion result, and (4) fewer parameters in the new model. The PPCNN model can be mathematically described using the following expressions:

(6) $$F_{ij}[n]=V_{F}\sum_{kl}M_{ijkl}Y_{kl}[n-1]+I_{ij}$$

(7) $$L_{ij}[n]=V_{L}\sum_{kl}W_{ijkl}Y_{kl}[n-1]+P_{ij}$$

(8) $$U_{ij}[n]=F_{ij}[n-1]+\beta_{ij}[n-1]L_{ij}[n-1]$$

(9) $$Y_{ij}[n]=\{\begin{array}{ll} 1 & U_{ij}[n]>E_{ij}[n]\\ 0 & \mathrm{otherwise} \end{array}$$

(10) $$E_{ij}[n+1]=e^{-\alpha_{E}}E_{ij}[n]+V_{E}Y_{ij}[n]$$

### 2.3. Implementation of PPCNN Model

**Figure 4:**
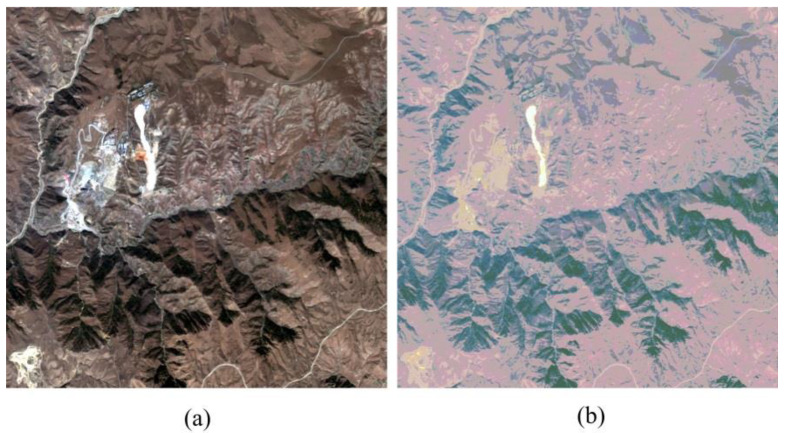
Structure of proposed PPCNN. (**a**) MS image. (**b**) *β* image.

Since the PPCNN model is mainly for the fusion tasks, the output part *Y* in the standard PCNN turns into the intermediate variable in the PPCNN model. Accordingly, the internal activity *U* represents the fusion result. Before the implementation of PPCNN is described, a couple of necessary symbols used should be defined. Symbol × indicates the multiplication between a constant and the matrix. Symbol • multiplies each element of one matrix with the corresponding one in the other matrix. Symbol ⊗ denotes the convolution between two matrices. Specifically, each neuron has its own *β_ij_* in the PPCNN model, designed as follows:

(11) $$C_{ij}[n]=\{\begin{array}{cc} \frac{\mathrm{Cov}(I,Pan)}{\mathrm{Cov}(Pan,Pan)} & \mathrm{if}\;Y_{ij}[n]=1\\ 0 & \mathrm{otherwise} \end{array}$$

(12) $$\beta_{ij}[n]=\{\begin{array}{cc} \frac{\mathrm{Std}(I)}{\mathrm{Std}(Pan)} & \mathrm{if}\;C_{ij}[n]>0\;\mathrm{and}\;Y_{ij}[n]=1\\ 0 & \mathrm{otherwise} \end{array}$$

where the symbol Cov(*X*, *Y*) stands for the covariance operation between *X* and *Y*. Std(*X*) indicates the standard deviation of *X*. *I* refers to the input multispectral image. *Pan* denotes the panchromatic image. An example of *β* image is shown in Figure 4.

The implementation procedure of PPCNN model includes the following steps:

1. Initializing matrices and parameters. *Y*[0] = *F*[0] = *L*[0] = *β*[0] = *U*[0] = 0, and *E*[0] = *V_E_*. The initial value of the iteration number *n* is 1. *P* is denoted as the spatial detail image. *I* and *P* are all normalized between 0 and 1. Other parameters (*V_F_*, *V_L_*, *V_E_ M*, *W*, and *α_E_*) are determined by experience based on different applications.
2. Compute *F*[*n*] = *V_F_* × (*Y*[*n* − 1] ⊗ *M*) + *I*, *L*[n] = *V_L_* × (*Y*[*n* − 1] ⊗ *W*)+*P*, and *U*[n] = *F*[*n* − 1] + *β*[*n* − 1]•*L*[*n* − 1].
3. If *U_ij_*[*n*] > *E_ij_*[*n*], then *Y_ij_*[*n*] = 1, else *Y_ij_*[*n*] = 0.
4. Update *E*[*n* + 1] = *e^-^^αE^* × *E*[*n*] + *V_E_* × *Y*[*n*].
5. Stop iteration until all neurons have been stimulated, and the internal activity *U* is the final fusion result, else let *n* = *n* + 1 and return to the Step (2).

### 2.4. Analysis of PPCNN Model

For the PPCNN model, Equation (8) can be rewritten below:

(13) $$U_{ij}[n]=V_{F}\sum_{kl}M_{ijkl}Y_{kl}[n-1]+\beta_{ij}[n-1]V_{L}\sum_{kl}W_{ijkl}Y_{kl}[n-1]+I_{ij}+\beta_{ij}[n-1]P_{ij}$$

The part *I_ij_* + *β_ij_* •*P_ij_* of the Equation (13) is similar to the traditional pansharpening method, except that *β_ij_* changes in each iteration. It means that the proposed model has the injection mechanism which is beneficial for pansharpening application. In addition, some good characteristics have been inherited from the standard PCNN in the new model, such as the phenomenon of the synchronous pulse emitting and the exponential attenuation of the threshold. As exhibited in Equation (13), the influences to the current neuron are not only from the external stimulus (*I* and *P*), but also from the neighborhoods, which guarantee the synchronous pulse emitting mechanism. In other words, the neurons with similar neighborhoods and stimuli will emit the pulses at the same time. Once the stimulus is a high-resolution image, the synchronous pulse emitting mechanism will make the interpretation of boundaries and textures of the object more accurate.

Another observation is that the threshold of the neuron decays exponentially according to Equation (10). Since the initial value *Y*[0] = 0 and *E*[0] = *V_E_*, *E_ij_* at the first iteration is

(14) $$E_{ij}[1]=e^{-\alpha_{E}}V_{E}=CV_{E}$$

Suppose that the neuron *ij* fires at the *n*_0_th iteration first, and fires again at the *n_l_*th iteration, where *l* is the iteration number of the firing event. When the iteration number *n* is less than *n*_0_, we have

(15) $$E_{ij}[n]=C^{n}V_{E}$$

Otherwise, while *n* is greater than *n*_0_, we get

(16) $$E_{ij}[n]=C^{n}V_{E}\sum_{l=0}^{L}\left(1+C^{-n_{l}}\right)=e^{n\ln C}\gamma_{L}$$

where *L* is the total firing times of the neuron *ij*.

According to Equation (9), the output pulse of the current neuron *ij* is generated when the threshold *E_ij_* is just less than the internal activity *U_ij_*. Thus, we have the firing time as follows:

(17) $$n\approx \ln(U_{ij}[n])/\ln(c)-\ln(\gamma_{L})/\ln(c)=a\ln(U_{ij}[n])+b$$

**Figure 5:**
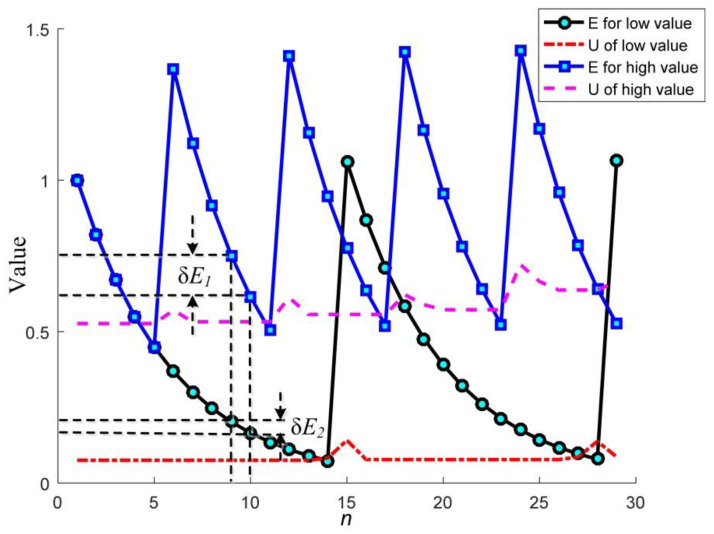
The exponential attenuation characteristic of the PPCNN model.

Since *U_ij_* is the accumulation of the external stimuli (multispectral and panchromatic images), Equation (17) obeys the Weber–Fechner law, i.e., there exists a logarithmic relationship between the firing time and the external stimuli. Therefore, the proposed model is coincide with the human visual perception. The exponential attenuation curve of the proposed model is shown in Figure 5. It is found that PPCNN processes the lower stimulus more precisely and the higher ones more coarsely. The characteristic is in accordance with the human visual system that people are very sensitive to contrast variation in the dark areas.

Compared with the detail injection mechanism of the most pansharpening methods and the pulse-emitting mechanism of standard PCNN model, the PPCNN model has the advantages of both by introducing the details injection mechanism into the standard PCNN model. In addition, as indicated in Equation (12), *β_ij_*[*n*] automatically changes based on the current firing environment in the iteration, which guarantees that the statistical calculations are computed among the neurons with the similar state. Furthermore, PPCNN is proved to coincide with the human visual system. These improvements are believed to be propitious for multispectral remote sensing image fusion.

## 3. PPCNN Based Pansharpening Approach

For pansharpening applications, there is a one-to-one corresponding relationship between the neurons and the input image. Thus a neuron of the PPCNN model is a mapping from a specific pixel in the remote sensing image. Before the model is applied, the input multispectral and the Pan images should be normalized between 0 and 1 so as to simplify the parameter settings of the network. Let *PI* and *MI_k_* be the input PAN image and the interpolated multispectral images, respectively. The normalized ones can be defined as:

(18) $$\varphi =\max(\max(MI_{1}),\cdots \max(MI_{K}),\max(PI))$$

(19) $$I_{k}=MI_{k}/\varphi k=1,\ldots K$$

**Figure 6:**
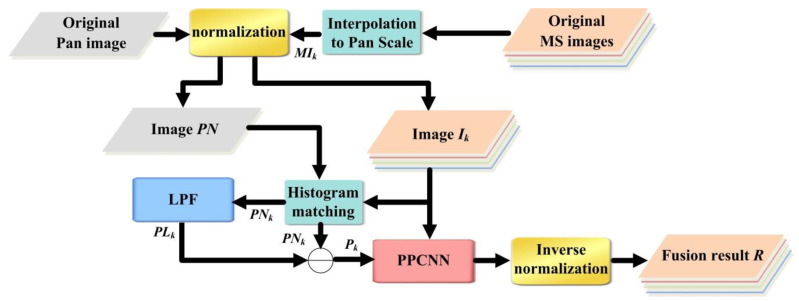
Procedure of PPCNN pansharpening approach.

(20) PN=PI/φ

where *k* indicates the *k*th band of the multispectral images, and the total band number is K. Symbol max indicates the maximum value of all pixels. Consequently, *I_k_* and *PN* are the corresponding normalized versions of *MI_k_* and *PI*. The architecture of the PPCNN pansharpening approach is shown in Figure 6, which consists of the following steps:

1. Interpolate the multispectral image to the PAN size by employing the even cubic kernel to acquire the input *MI_k_* [41].
2. Obtain *I_k_* and *PN* according to Equations (19) and (20).
3. Execute the histogram matching between *PN* and *I_k_* to obtain the matched version *PN_k_* of the PAN image.
4. Obtain the reduced-resolution version *PN_L_* of *PN* by using a low-pass filter, which obeys the modulation transferring function of the given satellite [42]. Calculate *P_k_* = *PN_k_* − *PN_Lk_,* where *k* = 1, …, K.
5. Set *k* = 1.
6. Update the internal activity *U_k_* after implementing the PPCNN model, where the external stimuli are *I_k_* and *P_k_*, respectively.
7. If *k* < K, let *k* = *k* + 1 and return to Step (6), otherwise, end the procedure.
8. Obtain the fusion result *R* by the inverse normalization transformation *R_k_* = *φ* × *U_k_*.

## 4. Experiments

In this section, several experiments are designed to evaluate the effectiveness of the proposed model. Obviously, fusing the remote sensing images with high spatial resolution is more difficult; thus, the experimental datasets were selected mainly on the high-resolution images. In this section, the evaluation criteria for assessing the fusion results are firstly briefly reviewed. Subsequently, several experimental datasets are prepared to testify the broad capability of the PPCNN model in the fusion applications of remote sensing images. Because the proposed PPCNN model is a laterally connected feedback network with no training, the parameter settings of PPCNN are also given in the section.

### 4.1. Quality Evaluation Criteria

The experimental results need to be evaluated with quantitative statistical criteria, which focus on both the spectral and spatial quality of the fusion results. Here, the measures used include the spectral angle mapper (SAM) [43], the relative dimensionless global error in synthesis (ERGAS) [44], the quaternion index (Q4) [45,46] and the spatial correlation coefficient (SCC) [47]. They are mathematically described as follows:

(21) SAM(x,y)=(1K)∑k=1Karccos(〈xk,yk〉/(‖xk‖⋅‖yk‖))

(22) ERGAS(x,y)=100r(1K)∑k=1K(RMSE2(xk,yk)/μ2(xk))

(23) $$Q(x,y)=\frac{4\mathrm{Cov}(x,y)\mu (x)\mu (y)}{\left(\mu^{2}(x)+\mu^{2}(y))(\sigma^{2}(x)+\sigma^{2}(y)\right)}$$

where the symbol < > represents the inner product and the symbol‖‖indicates the *l*_2_-norm. RMSE is the abbreviation of root mean square error. In addition, *r* stands for the spatial resolution ratio. The symbols *σ* and *μ* represent the variance and mean value, respectively.

SAM measures the global spectral accuracy, while ERGAS can represent the radiometric distortion between the ground-truth image and the fusion result. Q4 is used for overcoming the limitations of the root mean square error (RMSE) [9], which quantifies both spatial and spectral quality. SCC can evaluate the spatial correlation between two images. It should be noticed that the ideal values of SAM, ERGAS, Q4 and SCC are 0, 0, 1 and 1, respectively.

### 4.2. Datasets

**Figure 7:**
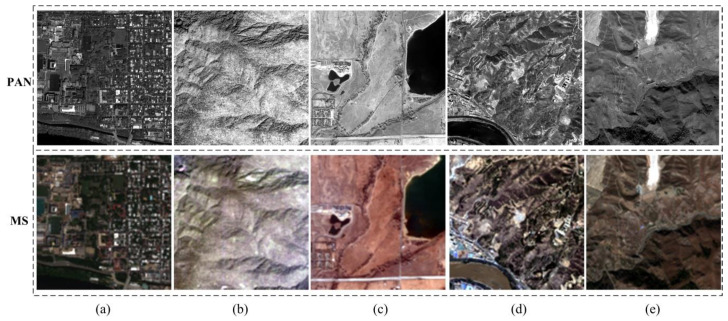
Datasets of pansharpening experiments. (**a**) Washington dataset. (**b**) Sichuan dataset. (**c**) Boulder dataset. (**d**) Lanzhou dataset. (**e**) Xinjiang dataset.

The performance of the PPCNN model has been well tested with five datasets. All the datasets represent different landscapes and are captured by four different high-resolution satellite sensors. The first dataset is captured by the WorldView-2 sensor over an urban area of Washington. The dataset mainly consists of buildings, woods, and the river. The second dataset is the landscape of the mountainous area of Sichuan province in China. It is captured by IKONOS-2 satellite sensor. The third dataset, which is captured from QuickBird satellite, indicates a suburban region of Boulder city in the United States. The fourth dataset, named Lanzhou dataset, is captured by the GF-2 sensor of China. The Lanzhou dataset represents a mountainous suburban area of Lanzhou city in northwest China, which is composed of the Yellow River, buildings and mountains. The fifth dataset is a dam area of Xinjiang province in China, which is also acquired using a GF-2 sensor. All the multispectral image of the datasets consists of four channels, i.e., green, blue, red, and near infrared bands. For convenient comparison, all the original images in the datasets are degraded based on the Wald’s protocol [48] so as to obtain the reference images. As a result, the original multispectral images can be used as the ground-truth images for accurate evaluation. Figure 7 shows the five datasets of the pansharpening experiments. The detailed parameters of all datasets are shown in Table 1.

### 4.3. Initialize PPCNN Parameters

**Figure 8:**
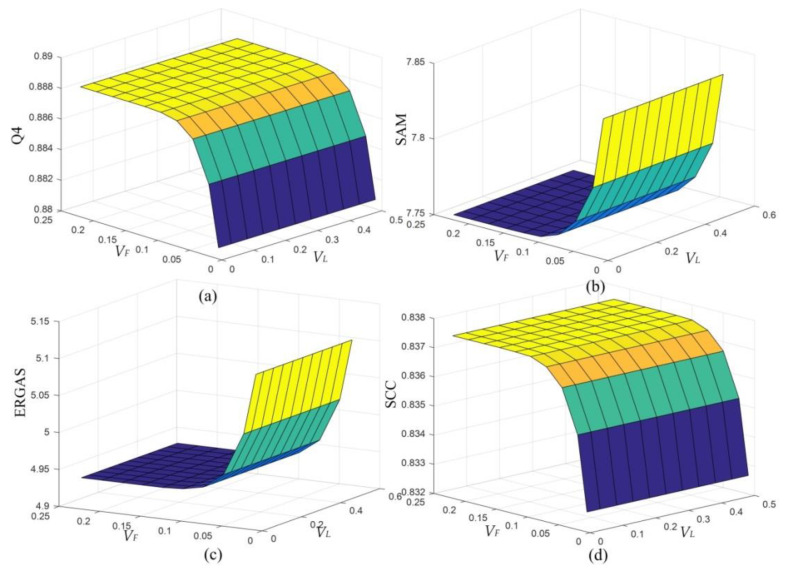
Analysis of the parameters *V_F_* and *V_L_*. (**a**) Q4. (**b**) SAM. (**c**) ERGAS. (**d**) SCC.

**Figure 9:**
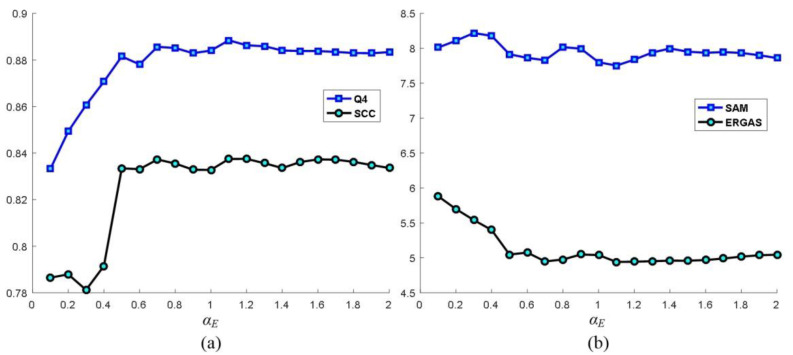
Analysis of the parameters *α_E_*.(**a**) Q4 and SCC. (**b**) SAM and ERGAS.

Since no training is needed in the PPCNN model, the settings of parameters and matrices are discussed here. Among which, linking synapses *M* and *W* with the matrix [0.707, 1, 0.707; 1, 0, 1; 0.707, 1, 0.707] are obtained by means of the Euclidean distance between two neurons. ***V_E_*** is set to be a large value to ensure that each neuron will be stimulated only once. The initial value of iteration number *n* is 1. The selection of ***V_F_*** and ***V_L_*** are analyzed in Figure 8. They are discussed as the PPCNN is carried out with the Washington dataset. Figure 8 gives the SAM, ERGAS, Q4 and SCC results of different ***V_F_*** and ***V_L_***, when ***α_E_*** equals to 1.1. It is found that ***V_L_*** offers little influence on the results. Another observation is that Q4 and SCC get the maximum values when the ***V_F_*** is larger than 0.1. SAM and ERGAS get the minimum values if the ***V_F_*** is larger than 0.1. Hence, the ***V_F_*** could be set as an arbitrary value when it is larger than 0.1. Figure 9 shows the SAM, ERGAS, Q4 and SCC curves of different ***α_E_***. We can see from Figure 9 that the optimal value of ***α_E_*** is 1.1.

## 5. Experimental Results and Discussions

In this section, the fusion experiments between the Pan image and the multispectral images are carried out. The performance of the proposed model will be well assessed, and the discussions will be made in detail. In the experiments, a couple of classical and state-of-the-art algorithms are presented as comparative methods, i.e., the GS method [17], the PCA method [15], the Brovey transform (BT) method [49], the IHS method [13], the ATWT method [20], the additive wavelet luminance proportional (AWLP) method [47], the CBD method [9], the revisited AWLP (RAWLP) method [50], the full scale regression (FSR) method [51] and the MOF method [22]. The pansharpening results obtained from the PPCNN algorithm and other methods are presented and discussed using five different datasets.

**Figure 10:**
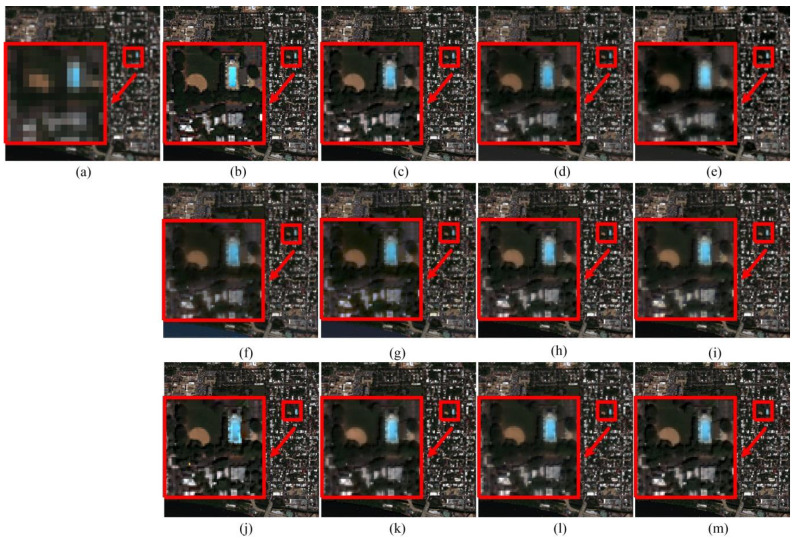
Pansharpening results of Washington dataset. (**a**) MS. (**b**) Ground-truth image. (**c**) PPCNN. (**d**) GS. (**e**) PCA. (**f**) BT. (**g**) IHS. (**h**) ATWT. (**i**) AWLP. (**j**) CBD. (**k**) MOF. (**l**) RAWLP. (**m**) FSR.

The experimental results with the Washington dataset are shown in Figure 10. Figure 10a shows the low-resolution multispectral image of the Washington dataset. Figure 10b gives the reference ground-truth multispectral image. The fusion result obtained from the proposed PPCNN method is shown in Figure 10c. The resulting fused multispectral images produced from GS, PCA, BT, IHS, ATWT, AWLP, CBD, MOF, RAWLP and FSR methods are shown in Figure 5d–m, respectively. From the Washington dataset obtained by WorldView-2, it can be noticed that the proposed PPCNN method, the ATWT method, the AWLP method, the CBD method, the MOF method, the RAWLP method and the FSR method perform better in spatial details and spectral preservation, while the contours in the PCA method and the BT method are blurred and not sharp enough. Wrong colors of small roof are obtained by the BT and the IHS methods. Table 2 provides the quantitative comparison results for the Washington dataset, where the best result is highlighted in bold. It can be found that the PPCNN approach performs better than the others with less spectral distortion and better detail preservation.

**Figure 11:**
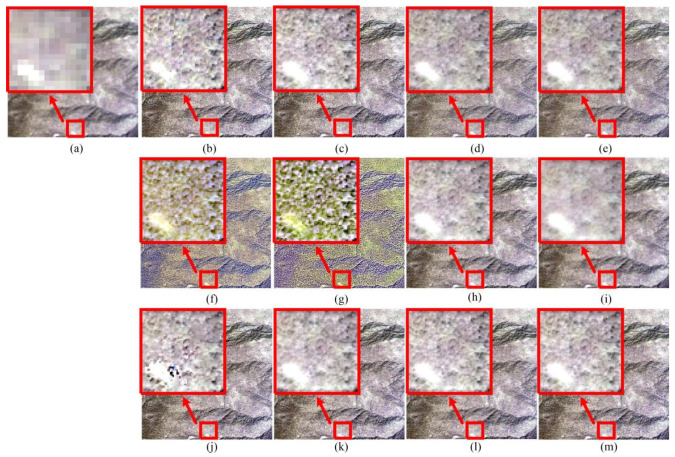
Pansharpening results of Sichuan dataset. (**a**) MS. (**b**) Ground-truth image. (**c**) PPCNN. (**d**) GS. (**e**) PCA. (**f**) BT. (**g**) IHS. (**h**) ATWT. (**i**) AWLP. (**j**) CBD. (**k**) MOF. (**l**) RAWLP. (**m**) FSR.

Figure 11 shows the pansharpening results of Sichuan dataset. As shown in Figure 11, the colors of the BT and IHS methods are not correctly synthesized. The AWLP method looks a little blurry. CBD method shows nice correct colors as a whole, but some other information computed by the method is redundantly added, especially in white area of Figure 11j. It has also been found that visual quality is generally acceptable for other seven methods. Table 3 demonstrates that the PPCNN method obtains the best fusion result for Sichuan dataset.

**Figure 12:**
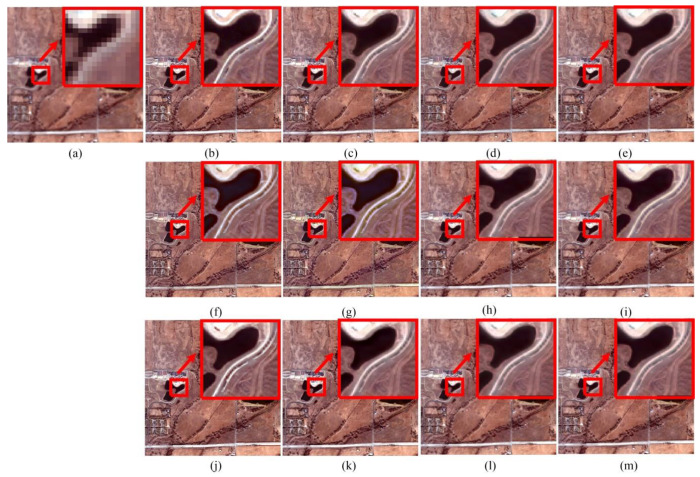
Pansharpening results of Boulder dataset. (**a**) MS. (**b**) Ground-truth image. (**c**) PPCNN. (**d**) GS. (**e**) PCA. (**f**) BT. (**g**) IHS. (**h**) ATWT. (**i**) AWLP. (**j**) CBD. (**k**) MOF. (**l**) RAWLP. (**m**) FSR.

From Figure 12, the experimental results for the Boulder dataset also show that the BT and IHS methods produce more spectral distortion, not only in the farmland and tree area, but also in the white roof area. For the Boulder dataset, wrong colors of small objects are also obtained using the CBD method, such as the fake red lines in the white roof. As shown in Table 4, the PPCNN method outperforms others in the quantitative comparison.

**Figure 13:**
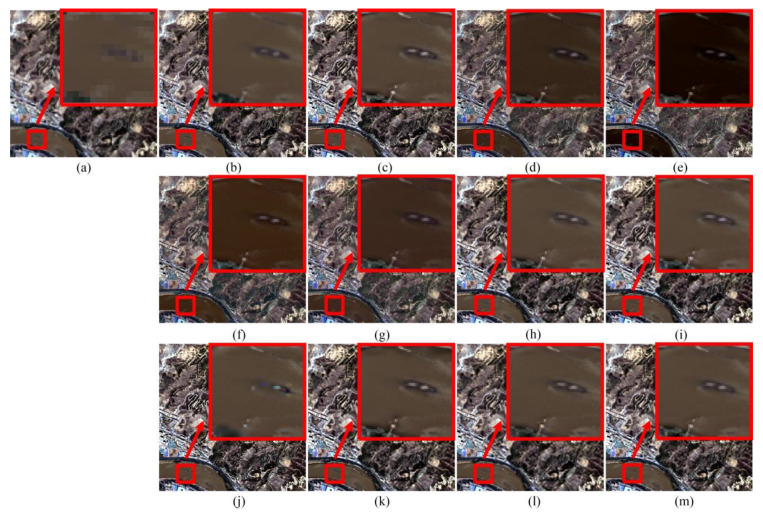
Pansharpening results of Lanzhou dataset. (**a**) MS. (**b**) Ground-truth image. (**c**) PPCNN. (**d**) GS. (**e**) PCA. (**f**) BT. (**g**) IHS. (**h**) ATWT. (**i**) AWLP. (**j**) CBD. (**k**) MOF. (**l**) RAWLP. (**m**) FSR.

For the experiments with the Lanzhou dataset, it is obvious from Figure 13 that the large spectral distortion is introduced by the GS, PCA, BT and IHS methods, particularly in the Yellow River area of Lanzhou city. In addition, part of the small island is missing in Figure 13j obtained by CBD methods. Another observation is that the tree belt looks a little blur in Figure 13j. Table 5 compares the PPCNN algorithm with others through the Lanzhou dataset. It shows that the proposed approach outperforms the others in the quantitative evaluation.

**Figure 14:**
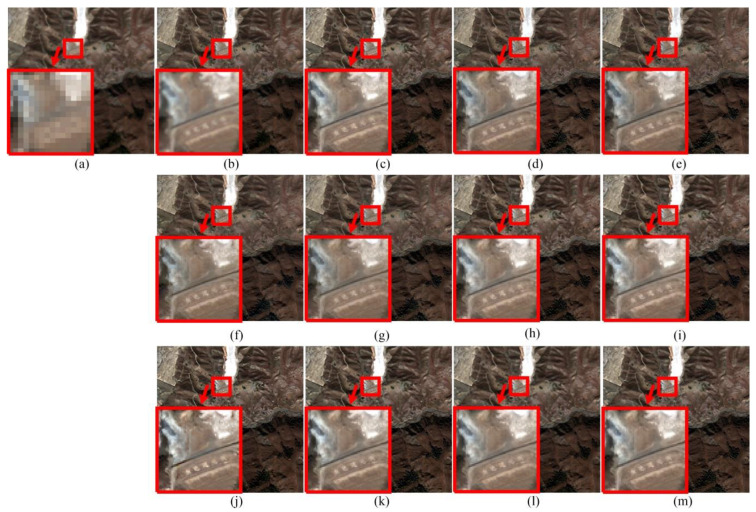
Pansharpening results of Xinjiang dataset. (**a**) MS. (**b**) Ground-truth image. (**c**) PPCNN. (**d**) GS. (**e**) PCA. (**f**) BT. (**g**) IHS. (**h**) ATWT. (**i**) AWLP. (**j**) CBD. (**k**) MOF. (**l**) RAWLP. (**m**) FSR.

Figure 14 shows the pansharpening results of the Xinjiang dataset. Since the Xinjiang dataset does not have a lot of high-resolution tiny textures, the visual quality is acceptable for all algorithms. However, Table 6 demonstrates that the PPCNN algorithm also performs the best.

**Figure 15:**
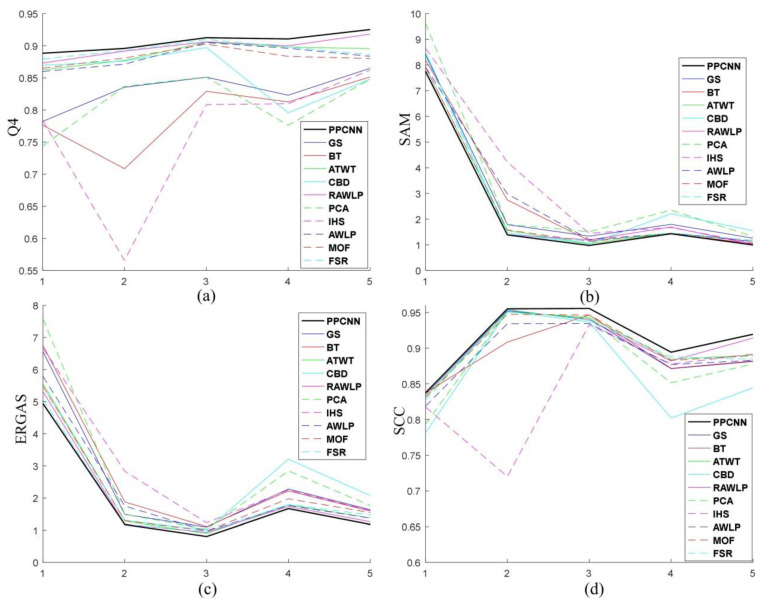
Comparison results of five datasets. (**a**) Q4. (**b**) SAM. (**c**) ERGAS. (**d**) SCC.

In conclusion, we tested the effectiveness of the PPCNN method with five datasets of different landscapes and sources. The experimental results of all datasets are summarized in Figure 15. In general, the results of the BT and IHS methods exhibited more spectral distortion. In some cases, GS and PCA methods are not good at spectral preservation either. More specifically, the contours of small objects obtained by these four methods and the AWLP method are sometimes blurred and not sharp enough. In addition, the CBD method produces spectral distortion in some small objects. Hence, the PPCNN, ATWT, MOF, RAWLP and FSR methods obtain good visual results. In greater detail, it can be noticed that the proposed PPCNN method produces images with better spatial detail quality and spectral quality than other methods according to the quantitative comparison.

## 6. Conclusions

The paper presents a novel PPCNN model and applies it to pansharpening approaches. The PPCNN model has two external stimuli rather than a single one in the standard PCNN, which guarantees it more convenient for fusion tasks. In addition, the internal activity of PPCNN is designed to have the function of details injection operation, which makes it easier to reserve the spectral fidelity. Five datasets with different characteristics, acquired by four different high-resolution sensors, were used to evaluate the model. The efficient performance of the PPCNN model is thoroughly examined through urban, suburban, mountainous and other complex landform datasets, which demonstrates that the PPCNN approach performs better with regard to both detail and spectral preservation.

In fact, the PPCNN model imitates the visual cortex neurons to transmit the input images to produce the synchronous electrical pulses with no training. On the other hand, deep learning models, such as CNN and RNN, imitate the mechanism of brain by training. If the output pulses of the PPCNN model are treated as the input of the CNN model, we believe that helpful results will be obtained. Consequently, the PPCNN based training model will be the focus of our future work. Another further interesting investigation looks toward the other image processing applications of PPCNN. We will investigate these problems in future work. 

## Figures and Tables

**Table 1:** Datasets for experiments.

Experiments	Datasets	Satellites	Size	Spatial Resolution	Region
Pan and MS	Washington dataset	WV-2	PAN: 2048 × 2048 MS: 512 × 512	0.5 m/2 m	Washington, D.C., USA
	Sichuan dataset	IKONOS-2	PAN: 2048 × 2048 MS: 512 × 512	1 m/4 m	Sichuan, China
	Boulder dataset	QuickBird	PAN: 2048 × 2048 MS: 512 × 512	0.7 m/ 2.8 m	Boulder, USA
	Lanzhou dataset	GF-2	PAN: 2048 × 2048 MS: 512 × 512	0.81 m/3.24 m	Lanzhou, China
	Xinjiang dataset	GF-2	PAN: 2048 × 2048 MS: 512 × 512	0.81 m/3.24 m	Xinjiang, China

**Table 2:** Comparison results for Washington dataset.

**Criteria**	**PPCNN**	**GS**	**PCA**	**BT**	**IHS**	**ATWT**
Q4	**0.8884**	0.7822	0.7438	0.7764	0.7841	0.8626
SAM (°)	**7.7527**	8.4126	9.6313	8.1936	8.6529	7.9265
ERGAS	**4.9416**	6.5617	7.5797	6.7480	6.6710	5.5633
SCC	**0.8375**	0.8349	0.7933	0.8372	0.8175	0.8317
**Criteria**	**AWLP**	**CBD**	**MOF**	**RAWLP**	**FSR**	
Q4	0.8600	0.8696	0.8651	0.8731	0.8795	
SAM (°)	7.9247	8.5065	7.9248	7.7967	8.3575	
ERGAS	5.7873	5.4875	5.4889	5.3147	5.0657	
SCC	0.8187	0.7812	0.8309	0.8369	0.8271	

**Table 3:** Comparison results for Sichuan dataset.

**Criteria**	**PPCNN**	**GS**	**PCA**	**BT**	**IHS**	**ATWT**
Q4	**0.8959**	0.8354	0.8368	0.7086	0.5658	0.8772
SAM (°)	**1.3857**	1.7843	1.7942	2.7454	4.2208	1.5599
ERGAS	**1.1769**	1.4884	1.4911	1.8771	2.8338	1.2873
SCC	**0.9552**	0.9525	0.9520	0.9085	0.7201	0.9509
**Criteria**	**AWLP**	**CBD**	**MOF**	**RAWLP**	**FSR**	
Q4	0.8714	0.8762	0.8810	0.8917	0.8926	
SAM (°)	2.9800	1.4627	1.5765	1.3983	1.3927	
ERGAS	1.7519	1.3027	1.2960	1.1865	1.1819	
SCC	0.9343	0.9515	0.9476	0.9540	0.9542	

**Table 4:** Comparison results for Boulder dataset.

**Criteria**	**PPCNN**	**GS**	**PCA**	**BT**	**IHS**	**ATWT**
Q4	**0.9126**	0.8511	0.8513	0.8291	0.8084	0.9057
SAM (°)	**0.9747**	1.3354	1.5113	1.1640	1.4448	1.0470
ERGAS	**0.8049**	1.0992	1.0346	1.1104	1.2317	0.8957
SCC	**0.9557**	0.9411	0.9411	0.9467	0.9328	0.9432
**Criteria**	**AWLP**	**CBD**	**MOF**	**RAWLP**	**FSR**	
Q4	0.9050	0.8972	0.9026	0.9062	0.9097	
SAM (°)	1.2062	1.0305	1.1257	1.1903	1.1528	
ERGAS	0.9731	1.0052	0.9763	0.9312	0.9441	
SCC	0.9346	0.9369	0.9465	0.9393	0.9392	

**Table 5:** Comparison results for Lanzhou dataset.

**Criteria**	**PPCNN**	**GS**	**PCA**	**BT**	**IHS**	**ATWT**
Q4	**0.9107**	0.8230	0.7763	0.8126	0.8097	0.8980
SAM (°)	**1.4304**	1.7992	2.3502	1.4307	1.6756	1.4545
ERGAS	**1.6697**	2.2794	2.8436	2.2208	2.2514	1.7911
SCC	**0.8944**	0.8713	0.8516	0.8717	0.8777	0.8844
**Criteria**	**AWLP**	**CBD**	**MOF**	**RAWLP**	**FSR**	
Q4	0.8961	0.7957	0.8835	0.9000	0.8982	
SAM (°)	1.4480	2.2117	1.4583	1.6917	1.4550	
ERGAS	1.7683	3.2100	1.9758	1.7373	1.7963	
SCC	0.8771	0.8021	0.8829	0.8822	0.8878	

**Table 6:** Comparison results for Xinjiang dataset.

**Criteria**	**PPCNN**	**GS**	**PCA**	**BT**	**IHS**	**ATWT**
Q4	0.9253	0.8650	0.8479	0.8514	0.8621	0.8956
SAM (°)	0.9940	1.2560	1.3230	1.0468	1.0672	1.1253
ERGAS	1.1784	1.6340	1.7619	1.5944	1.6054	1.3765
SCC	0.9195	0.8814	0.8778	0.8820	0.8918	0.8904
**Criteria**	**AWLP**	**CBD**	**MOF**	**RAWLP**	**FSR**	
Q4	0.8836	0.8479	0.8802	0.9181	0.8864	
SAM (°)	1.1277	1.5497	1.1718	1.0129	1.1674	
ERGAS	1.3733	2.0711	1.5450	1.2622	1.4706	
SCC	0.8830	0.8444	0.8896	0.9142	0.8847	

## References

[1] Huang B., Zhao B., Song Y. (2018). Urban land-use mapping using a deep convolutional neural network with high spatial resolution multispectral remote sensing imagery. Remote Sens. Environ..

[2] Fu H., Zhou T., Sun C. (2020). Object-based shadow index via illumination intensity from high resolution satellite images over urban areas. Sensors.

[3] Frick A., Tervooren S. (2019). A framework for the long-term monitoring of urban green volume based on multi-temporal and multi-sensoral remote sensing data. J. Geovis. Spat. Anal..

[4] Wang M., Zhang X., Niu X., Wang F., Zhang X. (2019). Scene classification of high-resolution remotely sensed image based on resnet. J. Geovis. Spat. Anal..

[5] Bovolo F., Bruzzone L., Capobianco L., Garzelli A., Marchesi S. (2010). Analysis of the effects of pansharpening in change detection on VHR images. IEEE Geosci. Remote Sens. Lett..

[6] Mohammadzadeh A., Tavakoli A., Zoej M.J.V. (2006). Road extraction based on fuzzy logic and mathematical morphology from pan-sharpened IKONOS images. Photogramm. Rec..

[7] Gašparović M., Jogun T. (2018). The effect of fusing Sentinel-2 bands on land-cover classification. Int. J. Remote Sens..

[8] Laporterie-Déjean F., Boissezon H., Flouzat G., Lefèvre-Fonollosaa M. (2005). Thematic and statistical evaluations of five panchromatic/multispectral fusion methods on simulated PLEIADES-HR images. Inf. Fusion.

[9] Vivone G., Alparone L., Chanussot J., Mura M.D., Garzelli A. (2015). A critical comparison among pansharpening algorithms. IEEE Trans. Geosci. Remote Sens..

[10] Amro I., Mateos J., Vega M., Molina R., Katsaggelos A.K. (2011). A survey of classical methods and new trends in pansharpening of multispectral images. EURASIP J. Adv. Signal Process..

[11] Thomas C., Ranchin T., Wald L., Chanussot J. (2008). Synthesis of multispectral images to high spatial resolution: A critical review of fusion methods based on remote sensing physics. IEEE Trans. Geosci. Remote Sens..

[12] Wang Z., Ziou D., Armenakis C., Li D., Li Q. (2005). A comparative analysis of image fusion methods. IEEE Trans. Geosci. Remote Sens..

[13] Tu T.M., Su S.C., Shyu H.C., Huang P.S. (2001). A new look at IHS like image fusion methods. Inf. Fusion.

[14] Carper W., Lillesand T., Kiefer R. (1990). The use of intensity-hue-saturation transformations for merging SPOT panchromatic and multispectral image data. Photogramm. Eng. Remote Sens..

[15] Psjr C., Sides S.C., Anderson J.A. (1991). Comparison of three different methods to merge multiresolution and multispectral data: Landsat TM and SPOT panchromatic. Photogramm. Eng. Remote Sens..

[16] Chavez P.S., Kwarteng A.Y. (1989). Extracting spectral contrast in Landsat thematic mapper image data using selective principal component analysis. Photogramm. Eng. Remote Sens..

[17] Laben C.A., Brower B.V. (2000). Process for Enhancing the Spatial Resolution of Multispectral Imagery Using Pan-Sharpening. U.S. Patent.

[18] Mallat S.G. (1989). A Theoryf Multiresolution Signal Decomposition: The Wavelet Representation.

[19] Nason G.P., Silverman B.W. (1995). The Stationary Wavelet Transform and Some Statistical Applications.

[20] Vivone G., Restaino R., Mura M.D., Licciardi G., Chanussot J. (2013). Contrast and error-based fusion schemes for multispectral image pansharpening. IEEE Geosci. Remote Sens. Lett..

[21] Burt P.J., Adelson E.H. (2003). The Laplacian pyramid as a compact image code. IEEE Trans. Commun..

[22] Restaino R., Vivone G., Mura M.D., Chanussot J. (2016). Fusion of multispectral and panchromatic images based on morphological operators. IEEE Trans. Image Process..

[23] Scarpa G., Vitale S., Cozzolino D. (2018). Target-adaptive cnn-based pansharpening. IEEE Trans. Geosci. Remote Sens..

[24] Masi G., Cozzolino D., Verdoliva L., Scarpa G. (2016). Pansharpening by convolutional neural networks. Remote Sens..

[25] Liu J., Ma J., Fei R., Li H., Zhang J. (2019). Enhanced back-projection as postprocessing for pansharpening. Remote Sens..

[26] Li H., Jing L., Tang Y., Ding H. (2018). An improved pansharpening method for misaligned panchromatic and multispectral data. Sensors.

[27] Aiazzi B., Alparone L., Baronti S. (2012). Twenty-Five Years of Pansharpening: A critical Review and New Developments. Signal and Image Processing for Remote Sensing.

[28] Wang N., Jiang W., Lei C., Qin S., Wang J. (2014). A robust image fusion method based on local spectral and spatial correlation. IEEE Geosci. Remote Sens. Lett..

[29] Ma Y., Zhan K., Wang Z. (2010). Applications of Pulse-Coupled Neural Networks.

[30] Wang Z., Ma Y. (2008). Medical image fusion using m-PCNN. Inf. Fusion.

[31] Zhu S., Wang L., Duan S. (2017). Memristive pulse coupled neural network with applications in medical image processing. Neurocomputing.

[32] Dong Z., Lai C., Qi D., Zhao X., Li C. (2018). A general memristor-based pulse coupled neural network with variable linking coefficient for multi-focus image fusion. Neurocomputing.

[33] Huang C., Tian G., Lan Y., Peng Y., Ng E.Y.K. (2019). A new pulse coupled neural network (pcnn) for brain medical image fusion empowered by shuffled frog leaping algorithm. Front. Neurosci..

[34] Cheng B., Jin L., Li G. (2018). Infrared and visual image fusion using LNSST and an adaptive dual-channel PCNN with triple-linking strength. Neurocomputing.

[35] Ma J., Xu H., Jiang J., Mei X., Zhang X. (2020). DDcGAN: A dual-discriminator conditional generative adversarial network for multi-resolution image fusion. IEEE Trans. Image Process..

[36] Ma J., Yu W., Liang P., Li C., Jiang J. (2019). FusionGAN: A generative adversarial network for infrared and visible image fusion. Inf. Fusion..

[37] Ma J., Liang P., Yu W., Chen C., Guo X. (2020). Infrared and visible image fusion via detail preserving adversarial learning. Inf. Fusion..

[38] Shi C., Miao Q.G., Xu P.F. (2013). A novel algorithm of remote sensing image fusion based on Shearlets and PCNN. Neurocomputing.

[39] Johnson J.L., Padgett M.L. (1999). PCNN models and applications. IEEE Trans. Neural Netw..

[40] Lindblad T., Kinser J.M. (2005). Image Processing Using Pulse-Coupled Neural Networks.

[41] Aiazzi B., Baronti S., Selva M., Alparone L. (2013). Bi-cubic interpolation for shift-free pan-sharpening. ISPRS J. Photogramm. Remote Sens..

[42] Aiazzi B., Alparone L., Baronti S., Garzelli A. (2006). MTF-tailored multiscale fusion of high-resolution MS and pan imagery. Photogramm. Eng. Remote Sens..

[43] Goetz A., Boardman W., Yunas R. Discrimination among semi-arid landscape endmembers using the Spectral Angle Mapper (SAM) algorithm. Proceedings of the Summaries 3rd Annual JPL Airborne Geoscience Workshop.

[44] Wald L. (2002). Data Fusion: Definitions and Architectures: Fusion of Images of Different Spatial Resolutions.

[45] Alparone L., Baronti S., Garzelli A., Nencini F. (2004). A global quality measurement of pan-sharpened multispectral imagery. IEEE Geosci. Remote Sens. Lett..

[46] Garzelli A., Nencini F. (2009). Hypercomplex quality assessment of multi/hyperspectral images. IEEE Geosci. Remote Sens. Lett..

[47] Otazu X., Gonzalez-Audicana M., Fors O., Nunez J. (2005). Introduction of sensor spectral response into image fusion methods. Application to wavelet-based methods. IEEE Trans. Geosci. Remote Sens..

[48] Wald L., Ranchin T., Mangolini M. (1997). Fusion of satellite images of different spatial resolutions: Assessing the quality of resulting images. Photogramm. Eng. Remote Sens..

[49] Gillespie A.R., Kahle A.B., Walker R.E. (1987). Color enhancement of highly correlated images. II. Channel ratio and “chromaticity” transformation techniques. Remote Sens. Environ..

[50] Vivone G., Luciano A., Andrea G., Simone L. (2019). Fast reproducible pansharpening based on instrument and acquisition modeling: AWLP revisited. Remote Sens..

[51] Vivone G., Rocco R., Jocelyn C. (2018). Full scale regression-based injection coefficients for panchromatic sharpening. IEEE Trans. Image Process..

